# Community assembly of root-colonizing arbuscular mycorrhizal fungi: beyond carbon and into defence?

**DOI:** 10.1093/ismejo/wrae007

**Published:** 2024-01-20

**Authors:** Adam Frew, Natascha Weinberger, Jeff R Powell, Stephanie J Watts-Williams, Carlos A Aguilar-Trigueros

**Affiliations:** Hawkesbury Institute for the Environment, Western Sydney University, Penrith, NSW 2751, Australia; Centre for Crop Health, University of Southern Queensland, Toowoomba, QLD 4350, Australia; Hawkesbury Institute for the Environment, Western Sydney University, Penrith, NSW 2751, Australia; Hawkesbury Institute for the Environment, Western Sydney University, Penrith, NSW 2751, Australia; School of Agriculture, Food and Wine, The Waite Research Institute, The University of Adelaide, Glen Osmond, South Australia 5064, Australia; Hawkesbury Institute for the Environment, Western Sydney University, Penrith, NSW 2751, Australia; Department of Biological and Environmental Sciences, University of Jyväskylä, Jyväskylä, 40014, Finland

**Keywords:** arbuscular mycorrhizal fungi, community assembly, carbon, plant defence, host selection

The arbuscular mycorrhizal (AM) fungi form symbiotic associations with the majority of terrestrial plants in a relationship estimated to be at least 470 million years old [1]. This symbiosis supported the terrestrialization of plants by facilitating their access to belowground nutrients, such as phosphorus. Today, AM fungi associate with most land plants where, as obligate symbionts, they rely entirely on their hosts for access to carbon as carbohydrates and lipids [2]. Yet the AM symbiosis does not exist in isolation. Simultaneous to AM fungal colonization, almost all plant hosts are subject to foliar damage from herbivores and pathogens. These antagonistic relationships are as ubiquitous as the AM symbiosis itself and have had significant impacts on the evolution and diversification of vegetation [3, 4]. Thus, this complex interplay among AM fungi, plants, and their herbivorous and pathogenic antagonists serves as a key driver in the ecological and evolutionary dynamics not only of the individual partners but also of global ecosystems.

A fundamental goal in ecology is to understand the underlying mechanisms of the assembly of communities. One of the most studied drivers of community assembly in ecology, particularly for plants and animals, is variation in resource availability [5]. In this context, community assembly is driven by distinct niches reflecting differences among species in either their preferred type of resources, their resource requirements, or how they acquire resources in time and space [6]. The principles that govern resource allocation and utilization in plant communities are, logically, considered applicable to AM fungi as well, underlining the universal relevance of resource availability in shaping ecological communities. Thus, it is not surprising that resource availability is one of the predominant mechanisms invoked to understand the assembly of AM fungi in plant roots from a local species pool. Dispersal factors, along with AM fungal preferences for certain soil characteristics and certain plant hosts, are all important in shaping the local species pool of AM fungi [7, 8], and ultimately the assembly of AM fungi in plant roots [9]. What is surprising, however, is the limited attention given to understanding how antagonistic interactions, such as herbivory or pathogen infection, determine the outcome of resource availability on AM fungal community assembly.

## Resource availability and arbuscular mycorrhizal fungi: carbon-for-nutrient exchange paradigm

As AM fungi are obligate biotrophs, carbon is their primary resource, and variation in its availability occurs when host plants alter the amount of carbon allocated to the fungi. Pioneering studies have observed that the carbon availability can be directly related to the amount of nutrients offered by the fungus to the plant [10, 11]. In this context, plants are often thought to ‘reward’ the most beneficial fungal symbionts, with the potential for adjustments of trade in resources depending on supply and demand from either partner [12]. Thus, for a particular host, the fungal taxa that are better at providing nutrients will be given access to more carbon. Evidence suggests that the ability of different fungal taxa to provide phosphorus to their host varies with environmental factors, including the physical, chemical, and biological characteristics of a soil [13]. Consequently, the most beneficial fungal symbiont that receives carbon from its plant host is highly context-dependent, allowing for diverse taxa to be favoured by the same host species under different environmental conditions.

There are instances where a host plant may limit carbon being delivered to the colonizing fungi, such as when soil fertility is high, or when photosynthesis is constrained. In these cases, we expect AM fungal taxa that have evolved the ability to thrive in low carbon settings to become dominant, regardless of their contribution to host plant nutrient uptake. Such conditions would arise when carbon fixation by the host plant is restricted by factors not directly related to the AM symbiosis, such as suboptimal light conditions or CO_2_. In those scenarios, the ability for these fungi to be successful may be related to their conservative production of mycelial biomass and spores, or they may use what carbon is available more efficiently when producing biomass, and thus represent a comparatively smaller carbon sink.

## Beyond carbon-for-nutrients: a role for plant defence in arbuscular mycorrhizal fungal community assembly

Although much discussion around the topic of AM fungal community assembly in roots is centred on the carbon for nutrient exchange, it is widely accepted that AM fungi provide their host plants with more services than simply nutrient acquisition. One key function that has garnered much attention is the ability for the AM symbiosis to enhance plant defences against insect herbivores and pathogens [14, 15]. Indeed, AM fungi have been shown to enhance a number of different defence mechanisms across a range of plant taxa [16, 17], often in ways that are unrelated to the nutrient outcomes for the host [18]. There is abundant evidence of the variation in the ability of AM fungal species to enhance plant defences [19, 20]. The variation in pathogen protection is assumed to be phylogenetically conserved among AM fungi [21–23], whereas less explicit consideration has been given to whether this is also the case for AM fungal-mediated protection from insect herbivores.

The diversity in the capacity for AM fungi to enhance plant defence raises the hypothesis that, during the evolution of AM fungi, host plants would preferentially reward species based on their ability to enhance plant defence, not just the ability to acquire physical resources for the host plant. This suggests there is evolutionary pressure for the fungi to retain these traits. Consequently, we would expect the assembly of AM fungi in the roots of plants that are challenged with pathogens or herbivory to preferentially be inclusive of fungi that can enhance their defence outcomes, and not just nutrient acquisition.

To our knowledge, the inclusion of carbon-for-defence has not formally been hypothesized as a major driver behind AM fungal community assembly in plant roots [24]. We understand the contributions of insect herbivores and pathogens to plant evolution and community assembly [4]. Although numerous studies have endeavoured to understand the drivers of AM fungal community assembly in plant roots, there is a dearth of research on pathogen or herbivory effects on AM fungal community assembly [25–27]. Here, we aim to stimulate discussion around these processes and their proximate and ultimate causes.

## Alternative mechanisms driving arbuscular mycorrhizal fungal community assembly: carbon constrained vs. defence directed

**Figure 1:**
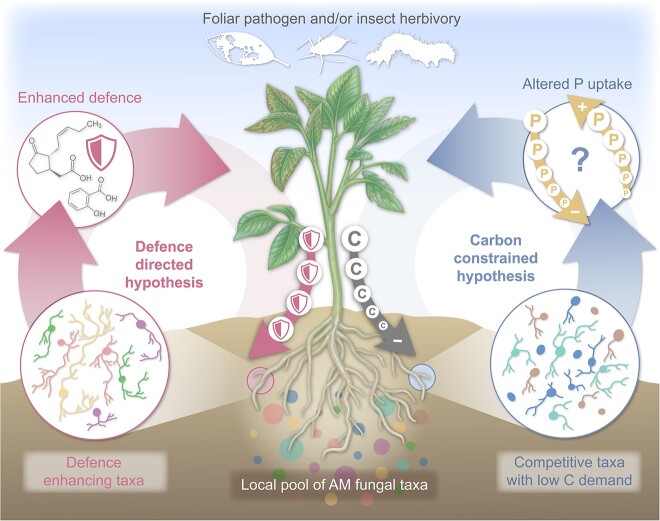
Conceptual figure illustrating the impact of insect herbivory and/or foliar pathogens on the assembly of root-colonizing arbuscular mycorrhizal (AM) fungal communities; the carbon constrained hypothesis proposes that reduced carbon (C) supply prompts competition among AM fungi, favouring those with lower carbon demands and/or strong carbon source competitiveness, this shift is likely to alter the overall phosphorus (P) uptake; the defence-directed hypothesis proposes that stressed plants selectively recruit AM fungi from the local pool of taxa with traits that enhance their defence mechanisms against herbivores and/or pathogens.

We propose two alternate mechanistic hypotheses behind AM fungal community assembly in roots of plants faced with aboveground herbivores or pathogens (Fig. 1). Both herbivory and pathogen infection put strain on the carbon budget of plants, which is expected to limit the carbon available to AM fungal symbionts [28]. Until recently, the data supporting this assumption have been equivocal. However, there is now growing evidence demonstrating that insect herbivory causes a reduction in the allocation of carbon to AM fungi [29, 30], although this has only been shown for single AM fungal species. For foliar pathogens, we expect a similar effect on carbon transfer from host to AM fungi. Although this has not yet been empirically demonstrated, to our knowledge, the fact that the same outcome has been observed with other biotic stressors (e.g. plant parasitic nematodes [31]) suggests that reduced C allocated to AM fungi in response to plant antagonistic interactions is likely to be a widespread phenomenon.

The **carbon constrained** hypothesis posits that the AM fungal community assembly is driven by the reduction in carbon availability as a result of antagonistic interactions. Competition among AM fungi would result in communities dominated by taxa that require less carbon or are stronger competitors. Such taxa may also provide nutrients (such as P) required by the host plant for growth or reproduction in response to antagonists. In this scenario, a plant may keep allocating carbon to a fungus it has previously invested in (which likely has already developed an extensive mycelium), to sustain nutrient uptake necessary for reproduction or compensatory growth [32].

On the other hand, our **defence-directed** hypothesis posits that when plant hosts are subjected to insect herbivory or pathogen attack, AM fungal communities would assemble in ways not attributable to changes in carbon dynamics alone. Instead assembly could be mediated by defence pathways being upregulated [33]. Here, the biochemical pathways associated with plant defence and mycorrhizal establishment are not independent. Expanding on this hypothesis further, certain fungi may even be preferentially selected by the host based on their ability to enhance plant defence, in a way that is analogous to mechanisms associated with rewards for fungi providing nutrients [10]. This version of our defence-directed hypothesis, based on reciprocal rewards during symbiosis, assumes the ability for a host plant to discriminate AM fungal taxa that elicit a stronger defence response than others in the species pool.

Certain AM fungal taxa elicit a stronger upregulation of phytohormonal defence pathways (e.g. jasmonic acid pathway) than others [34]. Thus, it is possible that hosts select based on their perception of signals generally known to play important roles in mycorrhiza establishment, such as defence-related molecules and phytohormones [35]. This mechanism would likely require plants to also perceive the physical location in the root system where those fungi are located so that carbon could be directed there [36], but see Verbruggen *et al.* [37]. For example, via fungal effector proteins that can be released and translocated into the plant nucleus in a temporal or spatial manner. Such effectors can initiate cascades leading to changes in hormone signalling, such as salicylic acid, which is important for long-distance signalling within plants [38]. Plants have developed mechanisms to recognize and respond to these effectors [39]. Indeed, recent research has identified several AM fungal-specific effectors, and it is predicted that more will be identified in the future [40, 41].

**Figure 2:**
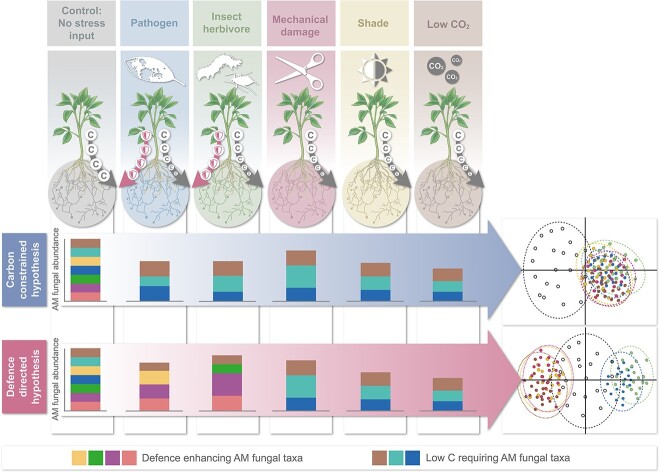
Plants face various factors which would be expected to reduce their carbon budget and limit carbon allocation to arbuscular mycorrhizal (AM) fungal symbionts in plant roots; given a hypothetical scenario where conspecific plants have the same available local pool of AM fungal taxa but are subjected to different factors that all reduce carbon availability, we would expect different AM fungal communities to be assembled depending on which hypothesis best explains the community assembly; if the defence-directed hypothesis best explains mechanisms of community assembly it would be expected that factors such as insect herbivory and pathogen attack would assemble communities distinct from those assembled when plants are subject to other carbon-reducing factors such as shade or low CO_2_; if the carbon constrained hypothesis best explains AM fungal community assembly, then the composition of communities in roots would be more similar to each other as they all are driven by carbon availability and competitive interactions; hypothetical AM fungal relative abundance stacked barplots are shown for each hypothesis and each scenario, where total barplot height refers to total AM fungal abundance and colour indicates more defence-enhancing taxa or taxa with low carbon requirements; hypothetical ordination plots are also shown to illustrate how the different communities might differ under each scenario depending on which hypothesis best explains assembly; each point on the ordination represents a community of AM fungi, the ellipses represent a 95% confidence interval; the colour of points and ellipses matches the scenarios under which those communities assemble and the black points and ellipses represent the controls, where plants are not subject to herbivores/pathogens or carbon limitation.

Given a hypothetical common pool of AM fungal taxa (i.e. that all individual host plants have equal access to the same AM fungal taxa), the carbon constrained hypothesis predicts that plants experiencing various forms of carbon limitation—such as shading, low CO_2_, or herbivory/pathogen-associated loss of photosynthetically active leaf area—would assemble comparatively similar AM fungal communities. This similarity would be driven exclusively (if not predominantly) by the reduction in carbon availability (Fig. 2), where differences in the carbon resource dynamics (i.e. amount and/or duration of reduced carbon etc.) would dictate community differences [42]. Consequently, heightened competition for this limited carbon resource would favour the emergence of fungal communities consisting of members with minimal carbon requirements or are highly competitive in this context.

However, under the defence-directed hypothesis, regardless of whether it is driven by specific selection for those fungi offering protection, or due to the complex biochemistry of attacked plants and the effects that has on symbiotic establishment, the identity of the trigger (insect herbivory, foliar pathogen, mechanical damage, shading, low CO_2_) would have strong impacts on the resultant AM fungal community composition (Fig. 2). Evidence suggests that contrasting molecular triggers among insects and non-specific mechanical wounding elicit different defence responses in plants [43, 44]. Additionally, even responses to true herbivory differ in their nature and/or strength depending on the feeding guild of the insect, e.g. piercing/sucking insects or chewing insects, and if there are single or multiple stimuli [43]. For plant pathogen responses, there is similar specificity, where changes in plant hormonal pathways can depend on pathogen identity. For example, both biotrophic pathogens and piercing/sucking insects are understood to activate salicylic acid-based defence responses in plants [45]. In contrast, necrotrophic pathogens and chewing insect herbivores mainly trigger jasmonic acid-associated defence responses [46]. Thus, it may be expected that mechanical tissue loss would result in community shifts that are more similar to chewing herbivory and necrotrophy infection than to, for example, shading or low CO_2_ availability, as these latter two would not be linked to plant defence and physical damage, but would still restrict the plant carbon budget. At its extreme (if different AM fungi can differentially alter plant defences against specific insect feeding guilds or pathogen lifestyles), AM fungal communities would differ depending on the type of insect herbivore (i.e. chewing, piercing) or pathogen lifestyle (i.e. necrotrophy, biotroph).

The processes outlined by the carbon constrained hypothesis and the defence-directed hypothesis may also operate concurrently where both carbon availability and the need for enhanced defence simultaneously influence fungal community assembly. For instance, a plant experiencing foliar damage from herbivory may prioritize the association with AM fungi that confer increased resistance; however, should the plant’s carbon resources become severely limited, the competitive dynamics among the fungal taxa would intensify. As a result, even a fungus that offers a defensive advantage could ultimately be excluded if it cannot sustain itself with reduced carbon or compete effectively with other fungi under these constraints. Host plants also simultaneously contend with herbivore or pathogen pressure in a carbon-limiting environment. For example, a plant subject to herbivory and shading may filter for fungi that enhance defence. Yet, as the plant’s photosynthetic capacity remains limited, this defence-oriented fungal community might reshuffle. Fungi with low carbon demands or those that can aggressively acquire the scarce carbon may come to dominate, potentially excluding even beneficial defence-enhancing fungi. This dynamic reflects the complex nature of AM fungal communities, where multiple factors such as carbon allocation and the necessity for defence coalesce, dictating the composition of the symbiotic partnerships.

## Implications and conclusions

Most microbial ecologists would agree that we are still a long way from having a complete understanding of microbial community assembly. This understanding comprises fundamental community ecology but is also essential for forecasting how communities will behave under different environmental scenarios. The AM symbiosis is important to plant productivity, plant community assembly, and has important roles in nutrient and carbon cycling, ultimately impacting ecosystem function. Although it is widely accepted that AM fungi are functionally diverse beyond nutrient uptake, the ecology and evolution of functions related to antagonistic interactions have received comparatively little attention [21]. For example, how conserved or variable functions are among AM fungal taxa, and how important this function is in their assembly, remains ambiguous. As the composition of the AM fungal communities which inhabit the root determines the outcome of the symbiosis for plants, it is important for us to know the drivers of and mechanisms behind the assembly processes. Until now, the role of defence on the assembly of AM fungi has been overlooked, despite clear importance of this function of the AM symbiosis.

There is ongoing interest and research in managing AM fungi for desired environmental outcomes, such as restoring degraded habitats, supporting ecosystem conservation, and promoting sustainable agriculture. Particularly within agricultural contexts, AM fungi are promoted not just for their nutrient acquisition functions but also for their plant defence capabilities. Often these endeavours can include an inoculation approach, whether with manipulated or ‘synthetic’ communities—typically comprising single or few taxa—or with native AM fungal communities that are translocated [47]. Expecting such efforts to be successful, particularly in the field, is naïve considering they are based on our currently incomplete understanding of how communities of AM fungi assemble in roots.

**Box 1. Outstanding questions**

- Is the fungal interspecific variation in AM-enhanced plant defence the results of selection or drift?
- Is the ability of AM fungi to enhance plant defence against insect herbivores phylogenetically conserved?
- How do benefits from AM fungal-mediated defences against herbivores and pathogens manifest in plant populations? For example, via improved growth and reproductive fitness of defended individuals compared to those under attack, or via other demographic patterns such as improved recruitment and reduced mortality?
- Is defence promotion associated with particular AM fungal traits, such as spore size, morphology of mycelium, biomass allocation, or metabolite expression?
- How much control do host plants have over carbon delivery to specific AM fungi all occupying the same root system with different effects on antagonistic interactions?
- To what extent do AM fungal species specialize in enhancing plant defences against different types of insect attacks, e.g. chewing versus piercing/sucking insects or necrotrophic versus biotrophic pathogens?
- What are the trade-offs between enhancing plant defences to other AM fungal functional (e.g. nutrient acquisition) and life history traits (size, growth rate, sporulation)? For example, does the ability to enhance host defences come at a cost of an ability to effectively forage and/or deliver resources (e.g. phosphorus) to a host?
- How would these potential trade-offs affect community assembly of AM fungi when hosts are faced with pathogen and/or herbivore pressure in an environment which is already carbon limiting for the host?
- What are the carbon demands of AM fungal species that specialize at enhancing defence mechanisms?
- In a carbon-for-defence market, would the economic dynamics be similar to those observed in a carbon-for-nutrient market? Specifically, would AM fungal taxa that are more effective at enhancing defence receive greater carbon allocations? Moreover, would the amount of carbon allocated fluctuate based on the intensity or nature of herbivore pressure?
- How variable is the reduction in carbon allocation to AM fungi in response to factors such as mechanical foliar damage or shading, and how does this variability impact AM fungal assembly?

## Data Availability

Data sharing is not applicable to this article, as no datasets were generated or analysed during the current study.
